# Ruscogenin ameliorates diabetic nephropathy by its anti-inflammatory and anti-fibrotic effects in streptozotocin-induced diabetic rat

**DOI:** 10.1186/1472-6882-14-110

**Published:** 2014-03-26

**Authors:** Hung-Jen Lu, Thing-Fong Tzeng, Shorong-Shii Liou, Sheng Da Lin, Ming-Chang Wu, I-Min Liu

**Affiliations:** 1 Department of Food Science, College of Agriculture, National Pingtung University of Science and Technology, Pingtung County, Taiwan; 2 Department of Pharmacy & Graduate Institute of Pharmaceutical Technology, Tajen University, Yanpu Township, Pingtung County, Taiwan

## Abstract

**Background:**

Ruscogenin is a major steroid sapogenin in the traditional Chinese herb Ophiopogon japonicus that have multiple bioactivities. Recent studies have demonstrated that ruscogenin is involved in down-regulation of intercellular adhesion molecule-1 (ICAM-1) and nuclear factor-κB (NF-κB) activation in anti-inflammatory pathways. We hypothesized that ruscogenin protects against diabetic nephropathy (DN) by inhibiting NF-κB-mediated inflammatory pathway. To test this hypothesis, the present study was to examine the effects of ruscogenin in rats with streptozotocin (STZ)-induced DN.

**Methods:**

Diabetes was induced with STZ (60 mg/kg) by intraperitoneal injection in rats. Two weeks after STZ injection, rats in the treatment group were orally dosed with 0.3, 1.0 or 3.0 mg/kg ruscogenin for 8 weeks. The normal rats were chosen as nondiabetic control group. The rats were sacrificed 10 weeks after induction of diabetes. Changes in renal function-related parameters in plasma and urine were analyzed at the end of the study. Kidneys were isolated for pathology histology, immunohistochemistry, and Western blot analyses.

**Results:**

Ruscogenin administration did not lower the levels of plasma glucose and glycosylated hemoglobin in STZ-diabetic rats. Diabetic rats exhibited renal dysfunction, as evidenced by reduced creatinine clearance, blood urea nitrogen and proteinuria, along with marked elevation in the ratio of kidney weight to body weight, that were reversed by ruscogenin. Ruscogenin treatment was found to markedly improve histological architecture in the diabetic kidney. Renal NF-κB activity, as wells as protein expression and infiltration of macrophages were increased in diabetic kidneys, accompanied by an increase in protein content of intercellular adhesion molecule-1 and monocyte chemoattractant protein-1 in kidney tissues. All of the above abnormalities were reversed by ruscogenin treatment, which also decreased the expression of transforming growth factor-β1 and fibronectin in the diabetic kidneys.

**Conclusions:**

Our data demonstrated that ruscogenin suppressed the inflammation and ameliorated the structural and functional abnormalities of the diabetic kidney in rats might be associated with inhibition of NF-κB mediated inflammatory genes expression.

## Background

It is increasingly apparent that not only is a cure for the current worldwide diabetes epidemic required, but also for its major complications, affecting both small and large blood vessels. These complications occur in the majority of individuals with both type 1 and type 2 diabetes [1]. Diabetic nephropathy (DN) is a serious complication in diabetes. Major typical morphological changes are the result of changes in the extracellular matrix (ECM). The ECM accumulation in DN results in mesangial expansion, tubulointerstitial fibrosis, and irreversible deterioration of renal function [2]. Even though previous studies have shown that ECM accumulation under diabetic conditions is attributable to hyperglycemia, hemodynamic changes, and local growth factors such as angiotensin II and transforming growth factor (TGF)-β1, the precise molecular and cellular mechanisms responsible for this have yet to be resolved [1]. Clarification of the pathogenesis of DN and development of novel and effective therapeutic strategies are therefore high priorities.

Although DN has not been traditionally considered an inflammatory disease, recent studies have shown that kidney inflammation is crucial in promoting the development and progression of DN [3,4]. It has demonstrated that macrophage infiltration into glomeruli is associated with the progression of DN [5]. Monocyte chemotactic protein-1 (MCP-1) and intercellular adhesion molecule-1 (ICAM-1), which make the monocytes/macrophages extravasculate from the blood-stream and attract to the kidney tissue, promote the development of DN [6,7]. Nuclear factor (NF)-κB, which regulates genes encoding proinflammatory mediators, involves in the inflammatory processes, and the activation of NF-κB and the transcription of certain proinflammatory chemokines have been demonstrated as the markers of progressive DN in patients [8]. These studies strongly suggest that NF-κB-mediated inflammatory processes represent a novel mechanism leading to DN. Therefore, it may be a novel therapy strategy for DN to reduce renal inflammation.

Ruscogenin (1β,3β,25R)-Spirost-5-ene-1,3-diol), first isolated from Ruscus aculeatus, has been reported to exert robust anti-inflammatory activities. It acts as an anti-elastase, decreases capillary permeability, and is widely used to treat chronic venous insufficiency and vasculitis [9-11]. In addition, ruscogenin is also a major steroidal sapogenin of the traditional Chinese herb *Radix Ophiopogon japonicus*, which has been clinically used to treat acute and chronic inflammatory and cardiovascular diseases [12,13]. Previous research has proved that the possible anti-inflammatory mechanism of ruscogenin was linked with the suppression of ICAM-1 expression in endothelial cells mainly through the inhibition of the NF-κB signaling pathway [14]. It was also found that ruscogenin significantly attenuated lipopolysaccharide (LPS)-induced acute lung injury model in mice, which possibly linked with inhibition of NF-κB activation [15]. Such activities of ruscogenin indicate its potential protection in diabetic kidney; however, there is no report about it until now.

Analyses of renal biopsies from type 1 and type 2 diabetic patients who develop DN indicate that inflammatory infiltrates are similar in both groups [16], which is consistent with studies in diabetic animal models [17,18]. To characterize its efficacy in the present study, ruscogenin was administered orally to streptozotocin-(STZ) induced diabetic rats for eight consecutive weeks. We present the results of this study and discuss the possible underlying action mechanism of this compound on diabetic renal lesions.

## Methods

### Animal models

Male Wistar rats (8 to 10 weeks of age, 200–250 g) were obtained from the Animal Center of National Cheng Kung University Medical College. To induce diabetes rats were given a single intravenous injection of 60 mg/kg streptozotocin (STZ; Sigma-Aldrich, Inc., St. Louis, Mo., USA). Animals were considered to be diabetic if they had plasma glucose concentrations of 350 mg/dl or greater, in addition to polyuria and other diabetic features. All studies were carried out two weeks after the injection of STZ. All animal procedures were performed according to the Guidelines for the Care and Use of Laboratory Animals of the National Institutes of Health (United States), as well as the guidelines of the Animal Welfare Act. The study was conducted with the approval of the Institutional Animal Care and Use Committee (IACUC) at Tajen University (approval number: IACUC 99–24; approval date: December 23, 2011).

### Treatment protocols

STZ-diabetic rats in the treatment group were dosed with 0.3, 1.0 or 3.0 mg/kg ruscogenin (≥ 98%; Chengdu Biopurify Phytochemicals Ltd., Chengdu, Sichuan, China; Cat. No. 472-11-7) in distilled water (1.5 ml/kg) by oral gavage once daily for eight weeks. The dosage regimen was selected based on a previous report demonstrating that ruscogenin at the indicated dosage regimen was potentially effective in inhibiting lipopolysaccharide-induced inflammation in mice [15]. Another group of STZ-diabetic rats was treated orally for eight weeks with 5 mg/kg/day rosiglitazone (purity ≥ 99.0%, Sigma-Aldrich, Inc.). The dose of rosiglitazone was based on studies with long-term treatment in STZ-diabetic rats [19]. A vehicle-treated groups of STZ-diabetic rats and normal rats were give 1.5 ml/kg distilled water by oral gavage over the same period. Animals had free access to standard rat diet (Harlan Teklad, Madison, WI, USA; Cat. No. 2018) and water throughout the entire treatment period. Treatment was continued even though the plasma glucose of STZ-diabetic rats was lower than 350 mg/dl during the eight-week treatment period. At the end of the eight-week treatment, the rats were weighed, and blood samples were collected from a tail vein. The evening prior to blood sample collection, animals were restricted to 3 g of chow (given at 18:00 h), which was consumed immediately, and thereafter had access to only water. The animals were transferred to metabolic cages (Shineteh Instruments Co., Ltd, Taipei, Taiwan), and urine was collected for 24 hours under a layer of toluene (to inhibit bacteria growth) and stored at 4°C for later analysis. Toluene had no detectable effect on the estimation of albumin and creatinine in the urine samples. Following urine collection, rats were sacrificed using an intraperitoneal injection of sodium pentobarbital (50 mg/kg).

The kidneys were dissected and rinsed with cold isotonic saline and weighed. The right kidney was stored immediately at −80°C in liquid nitrogen for biochemical determinations and Western blot analyses. Other kidney tissues were fixed in 10% neutralized formalin for histology.

### Blood sampling and analysis

Blood samples were centrifuged at 2 000 × g for 10 minutes at 4°C, and plasma was divided into aliquots for subsequent analyses. Plasma glucose concentration was determined using a diagnostic kit from BioSystem (Barcelona, Spain; Cat. No. COD12503). Serum creatinine (Scr) concentration was determined using a commercial assay kit purchased from Diagnostic Chemicals Limited (Connecticut, USA; Cat. No. 221–30). Blood urea nitrogen (BUN) was determined by kinetic reagent (Diagnostic Chemicals Limited, Cat. No. 283–30). Commercial enzyme-linked immunosorbent assay kits were used to quantify glycosylated hemoglobin (HbA_1c_) levels (Integrated Bio Ltd., Taipei, Taiwan; Cat. No. CSB-E08140r). All analyses were performed in accordance with the instructions provided by the manufacturers.

### Analysis of urine parameters

The 24-hour urine samples collected from each diabetic rat and age-matched control was centrifuged at 2 000 × g for 10 minutes. Urinary albumin concentrations were measured with the Nephrat II ELISA kit (Exocell, PA, USA; Cat. No. NR002). The concentration of creatinine in pooled urine samples was determined using a commercial assay kit (Diagnostic Chemicals Limited; Cat. No. 221–30). All analyses were performed in accordance with the manufacturer’s instructions. Creatinine clearance (Ccr) was calculated in individual rats using the relationship: Ccr = urine creatinine × (urine volume/plasma creatinine) × time [20].

### NF-κB activity measurement

Nuclear extracts of kidney from the above-mentioned groups were prepared using the nuclear extract kit (Active Motif, CA, USA; Cat. No. 40010). Twenty micrograms of nuclear extract were used for the determination of NF-κB activity with the TransAM® NF-κB p65 transcription factor assay kit (Active Motif; Cat. No. 40096) according to the manufacturer’s instruction.

### Renal cytokines determination

Renal tissue was homogenized in 10 mmol/L Tris–HCl buffered solution (pH 7.4) containing 2 mol/L NaCl, 1 mmol/L EDTA, 0.01% Tween 80, 1 mmol/L PMSF, and centrifuged at 9 000 × *g* for 30 min at 4°C [21]. The resultant supernatant was used for cytokine determination. ELISA kits for the determination of tumor necrosis factor-α (TNF-α) (Cat. No. ab46070), interleukin (IL)-6 (Cat. No. ab100772), and IL-1β (Cat. No. ab100768) were obtained from Abcam Inc. (Cambridge, MA, USA). Samples were assayed in duplicates according to manufacturer's instructions. The protein concentrations of kidney filtrate were determined using a Bio-Rad protein assay kit (Bio-Rad Laboratories, Japan) and bovine serum albumin (BSA) as a standard.

### Renal histological analysis

For histological analysis, the kidney was removed and embedded in paraffin to prepare 4-μm tissue slices. The tissue slices were stained with hematoxylin and eosin (H&E). The mesangial expansion index was scored in four levels from 0 to 3, with the index scores defined as follows [22]: 0, normal glomeruli; 1, matrix expansion occurred in up to 50% of a glomerulus; 2, matrix expansion occurred in 50 to 75% of a glomerulus; 3, matrix expansion occurred in 75 to 100% of a glomerulus. Scores were assigned for at least 30 glomeruli from kidney slices from each animal, and the means were calculated. Each slide was scored by a pathologist who was unaware of the experimental details.

### Immunohistochemistry

Formalin-fixed, paraffin-embedded kidney tissue sections were used for immunohistochemical staining. After deparaffinization and hydration, the slides were washed in Tris-buffered saline (TBS; 10 mmol/l Tris HCl, 0.85% NaCl, pH 7.2). Endogenous peroxidase activity was quenched by incubating the slides in methanol and 0.3% H_2_O_2_ in methanol. After overnight incubation with mouse monoclonal anti-monocyte/macrophage antibody (anti-ED-1) (Santa Cruz Biotechnology Inc. CA, USA; Cat. No. sc-59103), goat polyclonal anti-ICAM-1 antibody (Santa Cruz Biotechnology, Inc.; Cat. No. sc-1511), rabbit polyclonal nti-MCP-1 antibody (Abcam plc, Cambridge, UK; Cat. No. ab7202), mouse monoclonal anti-TGF-β1 antibody (Santa Cruz Biotechnology, Inc.; Cat. No. sc-52893), or rabbit polyclonal anti-fibronectin antibody (Abcam plc, Cat. No. ab2413) at 4°C, the slides were washed in TBS. Horseradish peroxidase-conjugated secondary antibody was then added, and the slides were incubated at room temperature for an additional 1 hour. The slides were washed in TBS, incubated with diaminobenzidine tetrahydrochloride as the substrate, and counterstained with hematoxylin. A negative control without primary antibody was included in the experiment to verify antibody specificity.

Sections were counterstained with haematoxylin for 15 seconds. Brownish yellow granular or linear deposits were interpreted as positive areas. Intraglomerular ED1-positive cells were counted in 30 glomeruli per animal at 400 × magnification by two independent observers with no prior knowledge of the experimental design [23]. The average number per glomerulus was used. Semi-quantitative assessments of the immunostaining of ICAM-1, MCP-1, TGF-β1, and fibronectin expression were scored using 4 levels, and an average value was obtained from analyses of more than 30 glomeruli per rat. The degree of ICAM-1, MCP-1, TGF-β1 and fibronectin expression in five rats from each group was graded as follows: 0, absent or < 25% staining; 1, 25% to 50% positive staining; 2, 50% to 75% positive staining; and 3, > 75% positive staining [22].

### Western blotting

Protein extraction of isolated kidney was performed as follows [24]. The sample was homogenized in ice-cold in 1 ml of hypotonic buffer A [10 mmol/l HEPES (pH 7.8), 10 mmol/l KCl, 2 mmol/l MgCl_2_, 1 mmol/l DTT, 0.1 mmol/l EDTA, 0.1 mmol/l phenylmethylsulfonylfluoride]. The cells were then lysed with 12.5 μl 10% Nonidet P-40. The homogenate was centrifuged, and supernatant containing the cytoplasmic extracts was stored frozen at −80°C. The nuclear pellet was resuspendedin 25 μl ice-cold nuclear extraction buffer. After 30 minutes of intermittent mixing, the extract was centrifuged, and supernatants containing nuclear extracts were secured.

Before immunoblotting, and the protein concentration of each tissue was determined using a Bio-Rad protein assay kit (Bio-Rad Laboratories, Japan) and BSA as a standard, to ensure equal loading among lanes. Nnuclear extracts (50 μg total protein) were separated on a 7.5-15% polyacrilamide gel and electophoretically transferred to nitrocellulose membrane. Membranes were blocked with 5% non-fat dry milk in Tris-buffered saline Tween (20 mmol/l Tris, pH 7.6, 137 mmol/l NaCl, and 0.1% Tween 20) for 3 h at room temperature, and incubated overnight at 4°C with the following primary antibodies: p-NF-κB p65 (Ser276) (Santa Cruz Biotechnology Inc.; Cat. No. sc-101749), NF-κB p65 (Cell Signaling Technology, USA; Cat. No. 4764,). The level of lamin A (Santa Cruz Biotechnology Inc.; Cat. No. sc-20680) was estimated for equal loading of nuclear sample. Three times after washing with Tris-buffered saline Tween 20 (TBST), incubation with appropriate horseradish peroxidase-conjugated secondary antibodies were performed for 1 h at room temperature. After three additional TBST washes, the immunoreactive bands were visualized by enhanced chemiluminescence (Amersham Biosciences, Buckinghamshire, UK) according to the manufacturer's instructions. Band densities were determined using ATTO Densitograph Software (ATTO Corporation, Tokyo, Japan). All experimental sample values were then expressed relative to this adjusted mean value. Tissue sections were sampled from 4 independent experiments.

### Statistical analysis

The results are presented as the mean ± standard deviation (SD) for each group of animals at the number (*n*) indicated. Statistical analysis was performed with one-way analysis of variance (ANOVA). The Dunnett range post-hoc comparisons were used to determine the source of significant differences where appropriate. The renal morphohistology and the morphologic analysis for PAS staining were analyzed statistically using the Kruskal-Wallis Test and Dunn’s Multiple Comparisons Test. Values of P < 0.05 were considered statistically significant.

## Results and discussion

DN is characterized by pathophysiological changes in glomerular hyperfiltration, renal hypertrophy, tubular function and then progress to proteinuria and reduction of glomerular filtration rate [2]. Accumulating evidence suggests that these clinical features can be linked, at least in part, to pathologic changes in the glomerular ECM [2]. STZ-diabetic rats showed an increase in 24-hour urine volume, accompanied by increase in urine protein excretion (Table 1). After eight weeks of ruscogenin or rosiglitazone treatment, 24-hour urine volume and 24-hour urine protein excretion for STZ-diabetic rats were markedly less than those of their vehicle-treated counterparts (Table 1). In addition, Scr and BUN levels in STZ-diabetic rats were obviously higher than in rats from the normal control group. These levels were effectively reduced in STZ-diabetic rats treated for eight weeks with ruscogenin relative to levels in their vehicle-counterparts (Table 1). In particular, increased Ccr in STZ-diabetic rats was obviously observed after eight weeks of ruscogenin or rosiglitazone treatment (Table 1). We also found that the kidney/body weight ratio was reduced by ruscogenin after 8 weeks treatment, suggesting that this compound may prevent diabetes-induced kidney enlargement. The previous study has been indicated that prevention of glomerular hypertrophy ameliorates the development of DN, including proteinuria and podocytopenia [25]. Thus, we demonstrated treatment with ruscogenin attenuated DN syndrome characterized by proteinuria and the loss of renal function in STZ-diabetic rats. Considering the effects of 3.0 mg/kg/day ruscogenin on the improvement of renal function in STZ-diabetic rats were closed to those produced by rosiglitazone, the kidney in STZ-diabetic rats receiving 3.0 mg/kg/day ruscogenin treatment was further isolated to delineate the potential underlying mechanisms.

**Table 1:** Body and kidney weights, index of renal hypertrophy, and the renal function-related parameters in experimental animals at the end of the eight-week treatment

	**Normal rats**		**STZ-diabetic rats**			
	**Vehicle**	**Vehicle**	**Ruscogenin (mg/kg/day)**			**Rosiglitazone (5 mg/kg/day)**
			**0.3**	**1.0**	**3.0**	
Body weight (BW) (g/rat)	374.88 ± 17.92^d^	214.84 ± 14.95^b^	237.3 ± 17.21^b^	261.98 ± 18.32^b,c^	291.64 ± 16.24^b,c^	315.18 ± 18.10^a,d^
Plasma glucose (mg/dl)	92.78 ± 7.32^d^	423.12 ± 16.22^b^	418.27 ± 15.34^b^	412.37 ± 17.41^b^	409.87 ± 14.68^b^	262.31 ± 13.71^b,c^
HbAlc (%)	4.79 ± 1.03^d^	14.32 ± 1.79^b^	14.10 ± 1.63^b^	13.92 ± 1.84^b^	13.85 ± 1.65^b^	9.63 ± 1.59^b,c^
Kidney weight (KW) (g/rat)	1.38 ± 0.17^d^	2.28 ± 0.15^b^	2.07 ± 0.13^b^	1.93 ± 0.21^b^	1.85 ± 0.19^a,c^	1.79 ± 0.18^a,d^
KW/BW ratio (%)	0.37 ± 0.07^d^	1.06 ± 0.08^b^	0.87 ± 0.09^b^	0.74 ± 0.06^b,c^	0.62 ± 0.04^a,d^	0.57 ± 0.05^a,d^
24-h urine volume (ml/day)	8.96 ± 1.76^d^	27.30 ± 2.95^b^	21.84 ± 3.11^b^	19.65 ± 2.43^b,c^	17.65 ± 3.05^a,c^	15.34 ± 2.62^a,c^
24-h urine protein (mg/day)	6.60 ± 2.57^d^	28.36 ± 3.95^b^	24.11 ± 3.25^b^	19.85 ± 2.57^,c^	14.82 ± 3.29 ^a,d^	10.26 ± 4.12^a,d^
Scr (μmol/l)	34.07 ± 5.26^d^	87.23 ± 7.96^b^	70.33 ± 5.83^b,c^	69.78 ± 4.92^b,c^	62.21 ± 5.61^b,c^	56.49 ± 6.92^b,c^
BUN (mmol/l)	6.51 ± 0.82^d^	18.85 ± 1.13^b^	15.68 ± 1.25^b,c^	14.27 ± 1.38^b,c^	12.66 ± 0.94^b,c^	9.71 ± 1.02^a,c^
Ccr (ml/min)	4.83 ± 0.63^d^	1.78 ± 0.71^b^	2.14 ± 0.56^b^	2.92 ± 0.61^b^	3.43 ± 0.59^a,c^	3.71 ± 0.74^d^

STZ-diabetic rats were dosed by oral gavage once per day for eight weeks with ruscogenin at the indicated dosage, or rosiglitazone. Normal or STZ-diabetic rats receiving vehicle treatment were given the same volume of vehicle (distilled water) used to prepare test medications. Values (mean ± SD) were obtained for each group of 8 animals. ^a^P < 0.05 and ^b^P < 0.01 compared to the values of vehicle-treated normal rats, respectively. ^c^P < 0.05 and ^d^P < 0.01 compared to the values of vehicle-treated STZ-diabetic rats, respectively.

**Figure 1:**
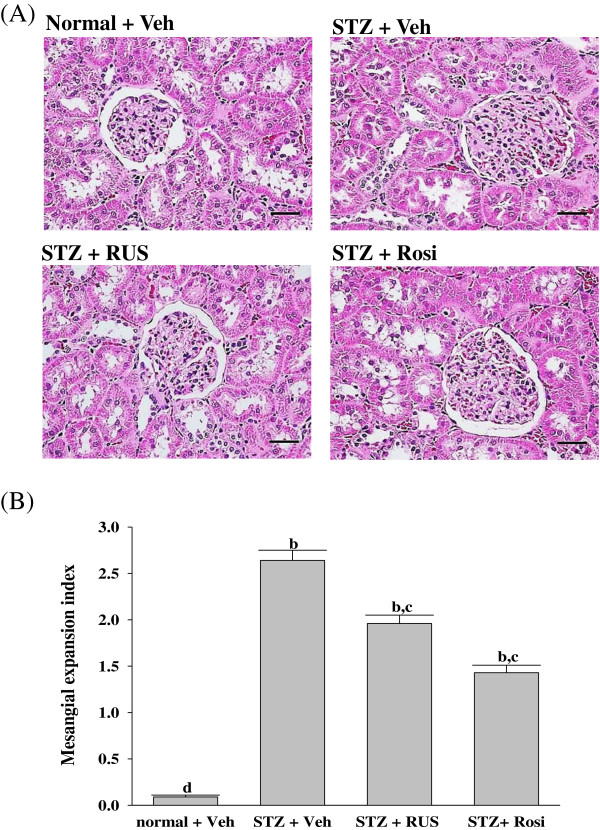
**Effects of treatments on the renal histology. (A)** Representative photomicrographs of H&E-stained kidney sections from STZ-diabetic rats treated for eight weeks with ruscogenin (RUS) or rosiglitazone (Rosi). STZ-diabetic rats were dosed by oral gavage once daily for eight weeks with 3 mg/kg RUS (STZ + RUS) or 5 mg/kg RGZ (STZ + Rosi). Normal (normal + Veh) or STZ-diabetic rats receiving vehicle treatment (STZ + Veh) were administered the same volume of vehicle (Veh) used to prepare test medications. Magnification bars = 100 μm **(B)** Results of quantification of the mesangial expansion index for each group. Values (mean ± SD) were obtained for each group of 5 animals. ^b^P < 0.01 compared to vehicle-treated normal rats (normal + Veh). ^c^P < 0.05 and ^d^P < 0.01 compared to vehicle-treated STZ-diabetic rats (STZ + Veh), respectively.

The STZ-diabetic rats showed focal mesangial matrix expansion compared to normal control rats (Figure 1A). Quantification of renal pathology showed that mean mesangial area was significantly increased in diabetic rats, however, treatment with rosiglitazone for 8 weeks markedly ameliorated mesangial expansion when compared with the untreated STZ-diabetic rats (Figure 1B). After eight weeks of treatment, enlargement of the mesangia in glomeruli was mildly attenuated in the STZ-diabetic rats treated with 3.0 mg/kg/day of ruscogenin. Quantitative analysis also showed that there was a marked decrease in the percentage of mesangial expansion in STZ-diabetic rats treated with 3.0 mg/kg/day ruscogenin compared with their vehicle-treated counterparts (Figure 1B). The kidney-protective effects of ruscogenin were further confirmed by the finding that ruscogenin treatment attenuated the structural abnormalities of DN.

**Figure 2:**
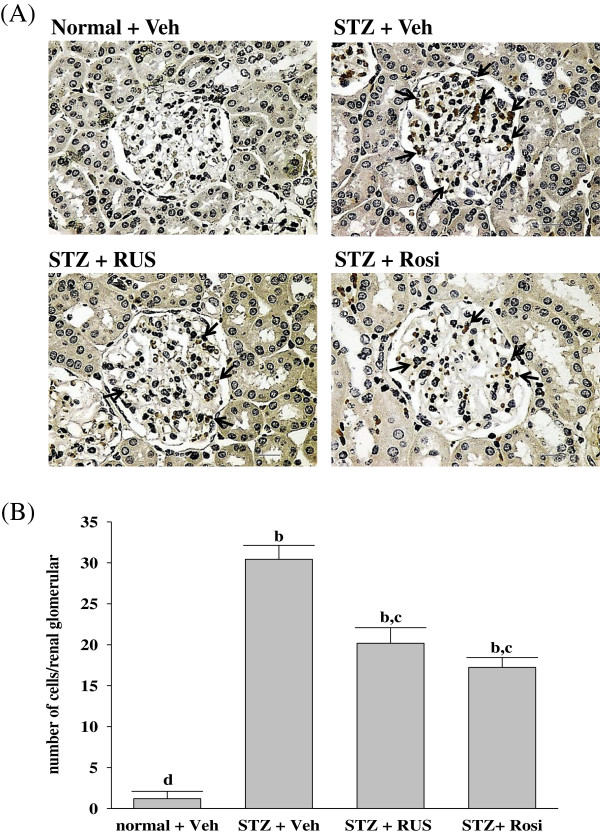
**Effects of treatments on macrophage infiltration. (A)** Immunohistochemical staining for macrophage (ED-1-positive) cells in the renal tissues of STZ-diabetic rats treated for eight weeks with ruscogenin (RUS) or rosiglitazone (Rosi). STZ-diabetic rats were dosed by oral gavage once daily for eight weeks with 3 mg/kg RUS (STZ + RUS) or 5 mg/kg RGZ (STZ + Rosi). Normal (normal + Veh) or STZ-diabetic rats receiving vehicle treatment (STZ + Veh) were administered the same volume of vehicle (Veh) used to prepare test medications. Arrows indicate positive areas. Magnification bars = 50 μm. **(B)** Quantified results are shown for number of macrophages (ED-1-positive cells). Values (mean ± SD) were obtained for each group of 5 animals. ^b^P < 0.01 compared to vehicle-treated normal rats (normal + Veh). ^c^P < 0.05 and ^d^P < 0.01 compared to vehicle-treated STZ-diabetic rats (STZ + Veh), respectively.

**Figure 3:**
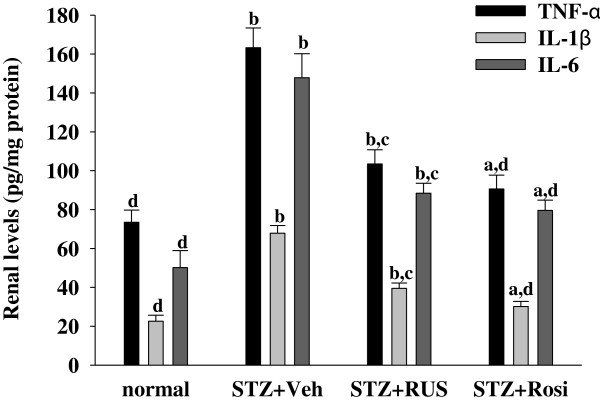
**Effects of treatments on cytokines levels in renal tissues of rats.** STZ-diabetic rats treated for eight weeks with ruscogenin (RUS) or rosiglitazone (Rosi). STZ-diabetic rats were dosed by oral gavage once daily for eight weeks with 3 mg/kg RUS (STZ + RUS) or 5 mg/kg RGZ (STZ + Rosi). Normal (normal + Veh) or STZ-diabetic rats receiving vehicle treatment (STZ + Veh) were administered the same volume of vehicle (Veh) used to prepare test medications. Values (mean ± SD) were obtained for each group of 8 animals. ^a^P < 0.05 and ^b^P < 0.01 compared to the values of vehicle-treated normal rats, respectively. ^c^P < 0.05 and ^d^P < 0.01 compared to the values of vehicle-treated STZ-diabetic rats, respectively.

Among the many potential pathogenetic mechanisms that are responsible for the development of diabetic kidney disease, an inflammatory mechanism has been suggested to be involved in the development of DN [3]. Macrophages are key inflammatory cells mediating kidney inflammation in experimental and human diabetes. In diabetes, macrophage accumulation and activation are associated with prolonged hyperglycemia, glomerular immune complex deposition, increased chemokine production, and progressive fibrosis [17,18]. Activated macrophages elaborate a host of proinflammatory, profibrotic, and antiangiogenic factors. Using accumulation of ED-1 as a marker of macrophage activation [26], we have demonstrated that increased macrophage activation in the glomeruli of kidney tissue from STZ-diabetic rats (Figure 2). In contrast, kidneys from control rats showed no significant macrophage infiltration (Figure 2). Treatment of STZ-diabetic rats with rosiglitazone or ruscogenin (3.0 mg/kg/day) for eight weeks caused a 33.8 ± 4.6% and 43.4 ± 3.9% reduction of macrophage influx, respectively, relative to that in their vehicle-treated counterparts (Figure 2). The renal expression of inflammatory cytokines such as TNF-α, IL-6 and IL-1β were demonstrated to increase in diabetes, contributing to the development of DN [27]. Along with the effects on macrophages, there was a reduction in the upregulated protein expression of TNF-α, IL-6 and IL-1β from kidneys of STZ-diabetic rats receiving ruscogenin (3.0 mg/kg/day) treatment (Figure 3). Thus, we believe that the anti-inflammatory effects of ruscogenin, through the inhibition of macrophage infiltration, might provide a renoprotective effect in the STZ- diabetic model.

**Figure 4:**
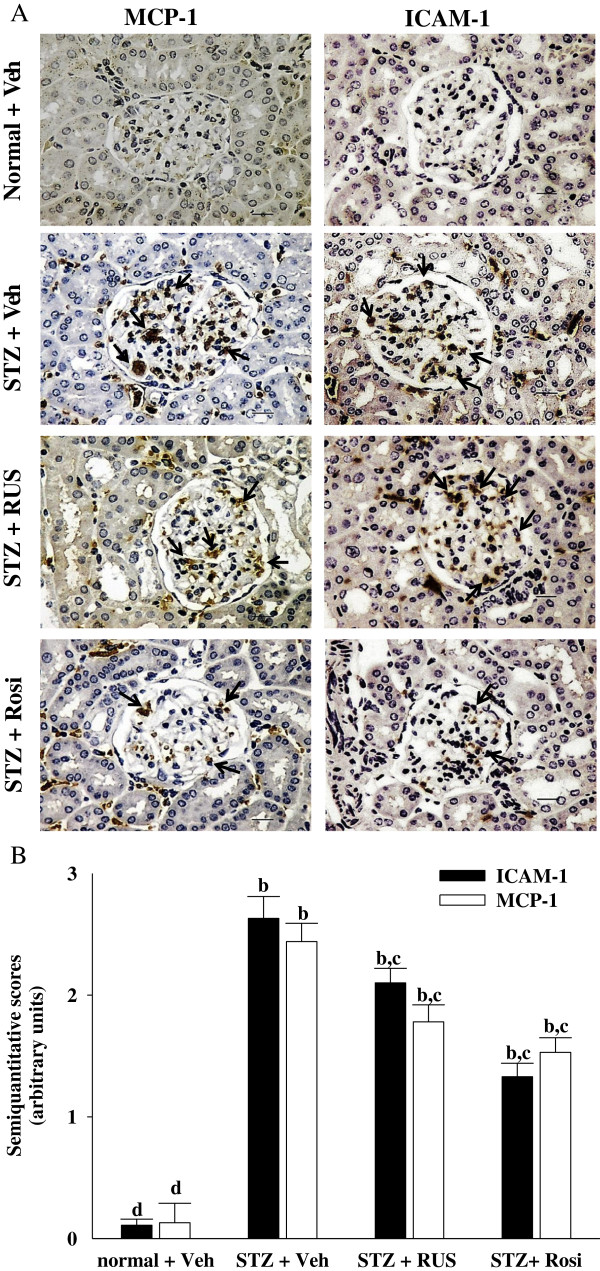
**Effects of treatments on protein expressions of MCP-1 and ICAM-1 in the renal tissues of rats. (A)** Immunohistochemical staining for MCP-1 and ICAM-1 in renal tissues of STZ-diabetic rats treated for eight weeks with ruscogenin (RUS) or rosiglitazone (Rosi). STZ-diabetic rats were dosed by oral gavage once daily for eight weeks with 3 mg/kg RUS (STZ + RUS) or 5 mg/kg RGZ (STZ + Rosi). Normal (normal + Veh) or STZ-diabetic rats receiving vehicle treatment (STZ + Veh) were administered the same volume of vehicle (Veh) used to prepare test medications. Arrows indicate positive areas. Magnification bars = 50 μm. **(B)** Semi-quantitative assessments of the immunostaining were scored using 4 levels, and an average value was obtained from analyses of more than 30 glomeruli per rat. Values (mean ± SD) were obtained for each group of 5 animals. ^b^P < 0.01 compared to vehicle-treated normal rats (normal + Veh). ^c^P < 0.05 and ^d^P < 0.01 compared to vehicle-treated STZ-diabetic rats (STZ + Veh), respectively.

ICAM-1 is a known important downstream inflammatory factor whose overexpression promotes inflammatory cells, including mononuclear macrophage infiltration into glomeruli and renal interstitium, as well as accelerates glomerular sclerosis in diabetes [6,7]. In addition to acting as a chemoattractant cytokine, MCP-1 may be involved in the inflammatory response by activating the macrophages from the circulation to the local kidney and then promote the expression of other proinflammatory cytokines to augment the accumulation of extracellular matrix [6,7]. The renal MCP-1 and ICAM-1 proteins were 2.6 and 2.4 fold higher in STZ-diabetic rats compared with normal rats, respectively. These increases were ameliorated by 30.4 ± 4.8% and 37.3 ± 5.2%, respectively, after eight weeks of treatment with rosiglitazone (Figure 4). Treatment of STZ-diabetic rats with 3.0 mg/kg/day ruscogenin for eight weeks resulted in a 20.2 ± 3.8% and 27.4 ± 4.9% reduction of renal MCP-1 and ICAM-1 protein expression, respectively, compared with that in vehicle-treated counterparts (Figure 4). The inhibitory effect of ruscogenin on MCP-1 and ICAM-1 may be partially due to the decreased infiltration of monocytes/macrophages. Therefore, a possible mechanism for preventing the progression of renal disease may involve the effect of ruscogenin to attenuate inflammation, by reducing the release of inflammatory mediators and/or inhibiting the expression of adhesion molecules in the diabetic kidney.

**Figure 5:**
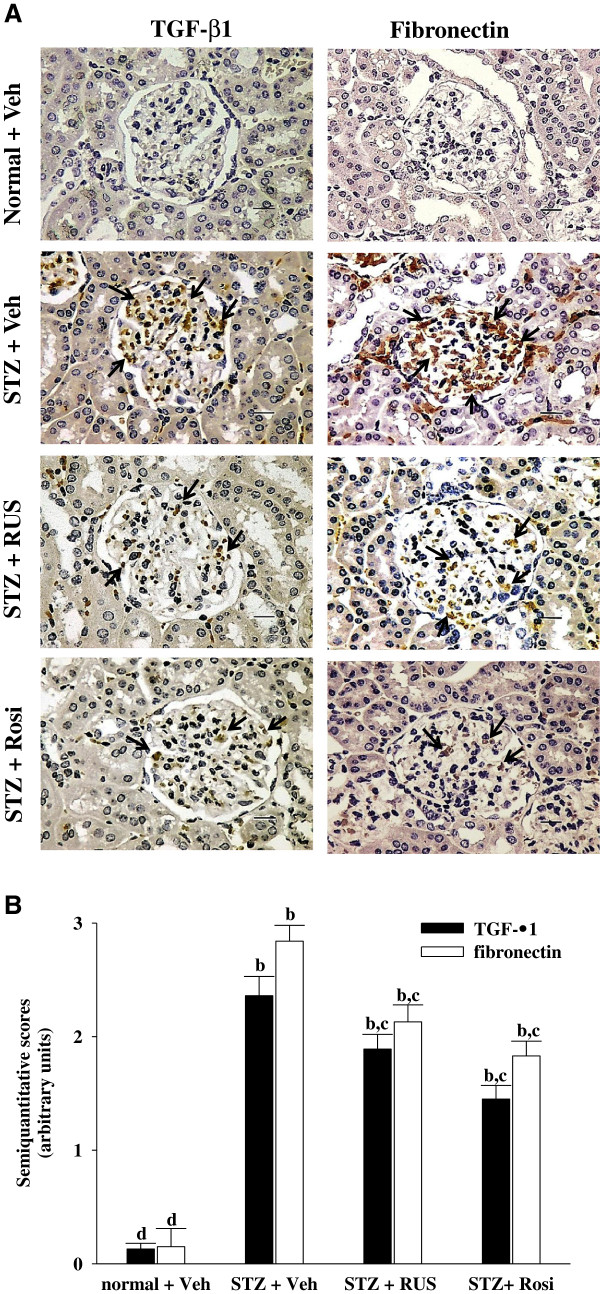
**Effects of treatments on protein expressions of TGF-β1 and fibronectin in renal tissues of rats. (A)** Immunohistochemical staining for TGF-β1 and fibronectin in renal tissues of STZ-diabetic rats treated for eight weeks with ruscogenin (RUS) or rosiglitazone (Rosi). STZ-diabetic rats were dosed by oral gavage once daily for eight weeks with 3 mg/kg RUS (STZ + RUS) or 5 mg/kg RGZ (STZ + Rosi). Normal (normal + Veh) or STZ-diabetic rats receiving vehicle treatment (STZ + Veh) were administered the same volume of vehicle (Veh) used to prepare test medications. Arrows indicate positive areas. Magnification bars = 50 μm. **(B)** Semi-quantitative assessments of the immunostaining were scored using 4 levels, and an average value was obtained from analyses of more than 30 glomeruli per rat. Values (mean ± SD) were obtained for each group of 5 animals. ^b^P < 0.01 compared to vehicle-treated normal rats (normal + Veh). ^c^P < 0.05 and ^d^P < 0.01 compared to vehicle-treated STZ-diabetic rats (STZ + Veh), respectively.

The profibrotic factor such as TGF-β1 is recognized as another important factor in the pathogenesis of DN by mediating inflammatory response, which aggravates extracellular matrix accumulation, as well as accelerates glomerular fibrosis in diabetes [28]. Thus, the inhibition of TGF-β1 expression benefits the treatment of diabetic kidney disease by alleviating matrix accumulation. Our results show that renal TGF-β1 protein levels were 2.4-fold higher in STZ-diabetic rats relative to normal rats; rosiglitazone treatment attenuated this increase by 30.1 ± 4.2% (Figure 5). The STZ-induced upregulation of TGF-β1 protein was reduced by 40.2% relative to that in vehicle-treated STZ-diabetic rats after eight weeks of treatment with ruscogenin (Figure 5). Furthermore, fibronectin, a major ECM protein, was induced in diabetic rats but ameliorated by ruscogenin (Figure 5). Therefore, we propose the anti-inflammatory, anti-fibrotic, and anti-hypertrophic effects of ruscogenin in DN may be attributable to the suppression of MCP-1 and ICAM-1 expression, by which inflammatory cell infiltration is abrogated, in turn ameliorating ECM accumulation.

**Figure 6:**
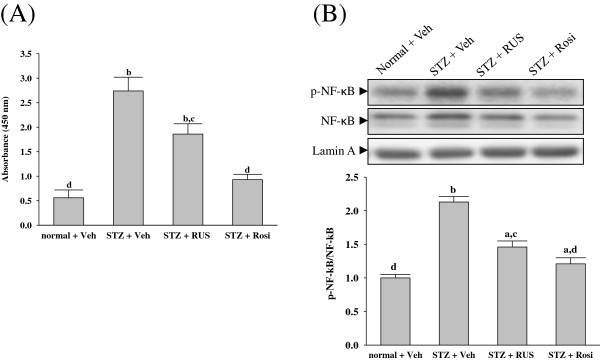
**Effects of treatments on NF-κB activity (A) and phosphorylated NF-κB (B) in renal tissues of rats.** STZ-diabetic rats treated for eight weeks with ruscogenin (RUS) or rosiglitazone (Rosi). STZ-diabetic rats were dosed by oral gavage once daily for eight weeks with 3 mg/kg RUS (STZ + RUS) or 5 mg/kg RGZ (STZ + Rosi). Normal (normal + Veh) or STZ-diabetic rats receiving vehicle treatment (STZ + Veh) were administered the same volume of vehicle (Veh) used to prepare test medications. The mean density values of p-NF-κB were expressed as ratios relative to that of NF-κB. The level of lamin A was estimated for equal loading of nuclear sample. Values (mean ± SD) were obtained for each group of 5 animals. ^a^P < 0.05 and ^b^P < 0.01 compared with vehicle-treated normal rats (normal + Veh), respectively. ^c^P < 0.05 and ^d^P < 0.01 compared to vehicle-treated STZ-diabetic rats (STZ + Veh), respectively.

NF-κB is a ubiquitous and well-known transcription factor responsible for regulating the expressions of genes that are involved in inflammatory pathways such as proinflammatory cytokines, chemokines and adhesion molecules [29]. Ruscogenin has been reported to have anti-inflammatory activity through inhibiting NF-κB activity [14,15]. However, it was unknown whether the inhibited effects of ruscogenin on inflammatory processes in DN might be mediated through inhibition of NF-κB activation. NF-κB undergoes phosphorylation on serine 276 in its p65 subunit and associates with surrounding chromatin components. It subsequently binds with DNA and promotes the transcription of proinflammatory cytokines, chemokines and adhesion molecules [29]. Thus, detection of the phosphorylated p65 subunit of NF-κB was effective for evaluating NF-κB activation [29]. Ruscogenin significantly inhibited NF-κB activation in kidney of diabetic rats, as evidenced by a decrease in NF-κB activity and downregulation of phosphorylated NF-κB (Figure 6). In the present study, ruscogenin exhibited anti-inflammatory effects through inhibiting NF-κB pathway, which was consistent with the previous study [14,15]. These results suggest that the inhibition of ruscogenin on renal inflammation in diabetic rats may be related to suppression of activation and overexpression of NF-κB.

It is widely known that activation of peroxisome proliferator-activated receptor (PPAR)γ attenuates the NF-κB-mediated transcriptional activation of proinflammatory genes [30]. Studies have also demonstrated that PPARγ agonist exerts a renoprotective effect through an anti-inflammatory mechanism in DN [19]. In the present study, the renoprotective effect of ruscogenin seems to be as effective as that produced by the standard drug rosiglitazone, an agonist of PPARγ. Unlike the effects of rosiglitazone, we found that ruscogenin administration did not inhibit the HgbA_1c_ nor lower hyperglycemia in STZ-diabetic rats (Table 1). Although ruscogenin be a ligand of PPARγ needs to be further investigated, the above results suggest that beneficial effect of ruscogenin in rats with DN is not mediated by its antihyperglycemic activity. This might be important, as we provided a data supporting that an anti-inflammatory intervention was effective in DN even without altering the blood glucose level.

Using a metabolism coefficient of 6.25 to convert the effective daily oral dose of ruscogenin for rat (3.0 mg/kg) into a clinical dose, assuming an average adult body weight of 60 kg [31], we estimated a daily oral dose of ruscogenin for humans to be approximately 32 mg. Due to different metabolism in humans and rats, the results come from rat studies cannot generalize to human. The placebo controlled human studies are required to find the usability of ruscogenin in human indication for DN.

## Conclusions

We have shown that the anti-inflammatory and anti-fibrotic effects of ruscogenin in DN may be attributable to prevention of NF-κB activation, by which inflammatory cell infiltration is abrogated, in turn ameliorating ECM accumulation. This study provides an important pharmacological and therapeutic basis for the treatment of DN.

## Competing interests

The authors declare that they have no competing interests.

## Authors’ contributions

HJL carried out the experimentation as part of PhD study. MCW contributed to study design, data interpretation and manuscript writing. TTF and SDL performed the experiments and analysis and participated to data interpretation. SLL supervised the work and evaluated the data. IML supervised the work, evaluated the data, manuscript writing and corrected the manuscript for publication. All authors read and approved the final manuscript.

## Pre-publication history

The pre-publication history for this paper can be accessed here:

http://www.biomedcentral.com/1472-6882/14/110/prepub
